# Knowledge, attitudes and perceptions regarding human papillomavirus among university students in Hail, Saudi Arabia

**DOI:** 10.7717/peerj.13140

**Published:** 2022-03-23

**Authors:** Farhan Alshammari, Kashif Ullah Khan

**Affiliations:** 1 Department of Pharmaceutics, College of Pharmacy, University of Hail, Hail, Saudi Arabia; 2 Department of Clinical Pharmacy, College of Pharmacy, University of Hail, Hail, Saudi Arabia

**Keywords:** Human papillomavirus, HPV vaccine, Knowledge, Attitude, Genital warts, University students, Saudi Arabia

## Abstract

**Background:**

Human papillomavirus (HPV) is a well-known cause of cervical cancer. The prevalence of HPV, insufficient preventive services, inadequate treatment access, socioeconomic conditions, certain cultural causes and values and opinions regarding cervical cancer have been established as factors contributing to the occurrence of cervical cancer in various parts of the world.

**Objective:**

To determine university students’ knowledge, attitudes and perceptions regarding HPV and its vaccine.

**Material and Methods:**

The present cross-sectional study included students enrolled at the University of Hail, Saudi Arabia. Data were collected from January to May 2020 using a previously validated 26-item questionnaire.

**Results:**

A total of 386 participants responded to the survey; the response rate was 80%. The majority of the respondents (63%) were male and 332 (86%) respondents were single among the overall study population. Most respondents were aged 21–25 years (75.6%), followed by 26–30 years (12.7%). In total, 130 (33.7%) respondents reported that they had heard of HPV before, while 174 (45.1%) reported that HPV infections are rare in Saudi Arabia. Furthermore, 102 (26.4%) respondents thought that HPV causes genital warts, while almost 29.5% believed that HPV infection is a sexually transmitted disease. Nearly 76.2% of the respondents did not believe that HPV infection can occur without symptoms. Moreover, 53.4% of the respondents stated that they did not know the health problems associated with HPV infection, while 148 (38.8%) stated that cervical cancer is a health problem associated with HPV infection. When asked about their understanding of the HPV vaccine, nearly 267 (62.2%) respondents believed that there is no vaccine for HPV, while 239 (61.9%) believed that the vaccine does not minimise the risk of cervical cancer. In addition, the respondents reported that they would be far more likely to get an HPV vaccine if recommended by their doctors [relative importance index (RII) = 0.745], followed by their friends (RII = 0.675).

**Conclusion:**

The present findings provide a clear understanding of university students’ knowledge, perceptions and attitudes regarding HPV; this information can be used to raise awareness by developing an effective educational strategy. However, further research with a larger sample size is recommended; such efforts would also aid in the development of educational services for various age ranges.

## Introduction

Human papillomavirus (HPV) infection is the most widespread viral infection of the reproductive tract (World Health Organization, 2016). HPV is a well-known cause of cervical cancer, in addition to cancers of the vulva, uterus, vagina, as well as genitals. It is also identified as causing cancer of oropharynx including the base of the tongue and tonsils (Forman et al., 2012). HPVs are natural pathogens that are passed from person to person through sexual contact. Many HPV genotypes have been categorised as medium- or high-risk ones based on their oncogenic ability in infected cells. There are more than 150 different types of HPV, with about 40 strains known to infect the genital area. HPV types 6, 11, 42, and 44 are non-oncogenic, although others (such as 16, 18, 31, 33, 35, 45, 51, 52, 58, 59, and 68) are known to be cancer-causing (Almughais, Alfarhan & Salam, 2018). Low-risk genotypes commonly cause anogenital warts, while high-risk genotypes are associated with genital cancers, particularly cervical cancers, and, to a lesser extent, endometrial and ovarian cancers (Almughais, Alfarhan & Salam, 2018; de Sanjose, Brotons & Pavon, 2018; Alsbeih, 2014). According to a survey conducted in Saudi Arabia, 2.3% of women in the general population are infected with high-risk HPV16/18 at given time (ICO/IARC Information Centre on HPV and Cancer, 2021). The HPV vaccine was first approved by the US Food and Drug Administration in 2006 (Zhang et al., 2016). Two HPV vaccines are widely available at present, and a couple of vaccines are in the pipeline (Yu et al., 2016). The HPV vaccine, which should be provided prior to the first sexual encounter, has been recommended by the World Health Organization (WHO) as the best way to avoid cervical cancer (WHO, 2013). Men have also been vaccinated against HPV in some countries because the available vaccines have been proven to be effective in avoiding anal pre-cancers and genital warts in both genders (WHO, 2013). According to global reports, 75% of sexually active individuals contract HPV over their lifespan (Matsuo et al., 2015). Consequently, in conservative societies, such as Saudi Arabia, where sexual partnerships are regulated by rigid social and religious laws, the prevalence of HPV is low (Turki et al., 2013; Al-Ahdal et al., 2014; AlObaid et al., 2014). However, in Saudi Arabia, an unprecedented rise in the occurrence of HPV infections has been recorded over the last couple of years, with up to 43% of cervical samples collected from healthy Saudi women being positive for HPV DNA, despite contradictory reports (Turki et al., 2013; Al-Ahdal et al., 2014; AlObaid et al., 2014; Alhamlan, Al-Qahtani & Al-Ahdal, 2015; Bondagji et al., 2013). Approximately 2.5 million cases of vulvar cancer and 13,200 cases of vaginal cancer were recorded globally in 2008 (de Martel et al., 2012). Nearly 60% of vulvar cancer cases and 68% of vaginal cancer cases are thought to occur in developing countries (de Martel et al., 2012). The HPV vaccine is also used in the national vaccination programmes of most developed countries, including Australia, Hungary and the United Kingdom (Yu et al., 2016). When the prevalence of HPV increased in 2014, 84 countries included the HPV vaccine in their national vaccination and immunisation programmes (Yu et al., 2016). The prevalence of HPV, insufficient preventive services, inadequate treatment access, socioeconomic conditions, certain cultural causes and values and opinions about cervical cancer have been established as factors contributing to the occurrence of cervical cancer in women living in various parts of the world (Onon & Kitchener, 1999; Yao et al., 2017; Guan et al., 2012; Richardson, Tota & Franco, 2011). According to the Ministry of Health (MOH) of Saudi Arabia, HPV infections are a significant cause of cancer, particularly cervical cancer, worldwide. Furthermore, in 2014, the MOH of Saudi Arabia reported that immunisations help protect against cancer-causing viruses, such as HPV (MOH, 2014). In Saudi Arabia, there is a scarcity of data on public awareness and comprehension of HPV. Furthermore, few public health programs are geared at increasing young people’ awareness of the symptoms, causes, and preventative actions associated with HPV infection. The major source of HPV awareness among the young population may be social media and some web blogs. There are currently no national efforts in Saudi Arabia to promote awareness about sexually transmitted diseases (STDs) and HPV among adults. As a result, the purpose of this study was to look at university students’ knowledge, attitudes, and perception regarding HPV. The study’s findings are meant to give a baseline understanding of young individuals who may be at risk of HPV infection, allowing public health officials to act if necessary. Studies have shown that social, cultural, and economic aspects are linked to the uptake of HPV vaccines throughout time (Bruni et al., 2016; Gallagher, LaMontagne & Watson-Jones, 2018). In Saudi Arabia, there is limited data on the knowledge, attitudes, and habits of people of various ages and genders when it comes to HPV and its vaccine. As a result, this study looked into the knowledge, attitudes, and practices of bachelor, master, and exchange students at the University of Hail regarding HPV infection and its vaccine. This study can serve policy implications and decision-makers in that they should be aware of the overall level of knowledge among health-care workers and implement educational interventions to address information gap and assist health practitioners in consistently recommending the anti-HPV vaccine.

## Materials and Methods

The present cross-sectional study included students enrolled at both male and female campuses at the University of Hail, Saudi Arabia, the only public sector tertiary institution in the region. Data were collected from January to May 2020. To assess the university students’ knowledge regarding HPV and its vaccine, a convenience sampling approach was adapted and a self-administered, previously validated 26-item questionnaire was used with some modifications (Khan et al., 2016). Convenience sampling was used because the target population meet certain practical criteria, such as ease of access, geographic closeness, availability at a specific time, or willingness to participate, are included for the study (Etikan, Musa & Alkassim, 2016). The student sample was distributed mainly into Health sciences and non-health sciences comprising of different schools and faculties where students were registered. Moreover, the sample was also distributed among the students educational level where it was categorized into bachelors, masters and doctoral level students.

### Study tool

A previously validated 26-item questionnaire was used in the present study with slight modifications with the approval of the authors. A panel of five academic experts was approached for validating the content of the questionnaire; the content was then validated and approved by the panel experts after slight changes to overcome the desirability bias and also enough time was given to the participants for completion of the questionnaire tool to avoid any recall bias (Althubaiti, 2016). A 26-item questionnaire was piloted among 20 respondents once content validity was completed. For these 20 respondents, the reliability scale was used, and the alpha value was determined to be 0.878, indicating that the instrument is suitable for meeting the study’s objectives (Khan et al., 2016) (Appendix 1).

### Contents of the questionnaire

The questionnaire consisted of five sections. The first section had six questions for collecting the respondents’ demographic data (Table 1). The second section aimed to assess the respondents’ general understanding of HPV. For the respondents’ ease, a nominal scale (yes/no) was used to provide the responses (Table 2). The third section of the questionnaire consisted of three key items designed to test the respondents’ awareness of the effects of HPV, its prevention and its dissemination (Table 3). The fourth section consisted of five questions and used a nominal scale (yes/no) to assess the respondents’ experience with the interpretation of HPV vaccines. The last section consisted of three key questions and used a five-item Likert scale to record the respondents’ opinions about HPV vaccination (Tables 4 and 5).

**Table 1:** Demographics of respondents who participated in the survey (N = 386).

Demographics	*N* (%)
Gender	
Male	243 (63.0%)
Female	143 (37.0%)
Marital Status	
Married	54 (14.0%)
Single	332 (86.0%)
Age	
Mean SD 24 ± 7.1 years	
Median 22 years (Range 18–35 years)	
<20 years	25 (6.5%)
21–25 years	292 (75.6%)
26–30 years	49 (12.7%)
>30 years	20 (5.2%)
Education level	
Bachelor’s	351 (90%)
Master	21 (5.4%)
PhD	14 (3.6%)
Field of Study	
Health sciences	192 (49.7%)
Non-health sciences	194 (50.3%)
Course Registered	
Pharmacy (Pharm-D)	102 (26.6%)
Bachelor of Medicine and Bachelor of Surgery	21 (5.4%)
Bachelor of Dental Studies	16 (4.1%)
Engineering	45 (11.7%)
Education Studies	68 (17.6%)
Arts & Humanities	32 (8.3%)
Social Sciences	15 (3.9%)
Biological Sciences	7 (1.8%)
Physiotherapy	7 (1.8%)
Others	73 (18.9%)

**Table 2:** Respondents’ general knowledge regarding human papillomavirus (N = 386).

Q. No	Statement	Yes	No	Gender	Field
7	Before taking this survey, had you ever heard of HPV (human papillomavirus)?	130 (33.7%)	256 (66.3%)	0.566* [0.360–0.891]	0.364* [0.238–0.574]
8	HPV is sexually transmitted?	114 (29.5%)	272 (70.5%)	0.700 [0.445–1.099]	1.859* [1.189–2.905]
9	HPV infections are rare in Saudi Arabia?	174 (45.1%)	212 (54.9%)	0.891 [0.588–1.349]	0.825 [0.552–1.234]
10	HPV causes cervical cancer?	96 (24.9%)	290 (75.1%)	0.687 [0.428–1.103]	1.777* [1.109–2.847]
11	HPV may infect both, men and women?	153 (39.6%)	233 (60.4%)	0.989 [0.647–1.511]	1.472 [0.977–2.218]
12	The incidence of HPV is highest among Women in their 20’s and 30’s?	81 (21.0%)	305 (79.0%)	0.939 [0.566–1.559]	1.525 [0.929–2.505]
13	HPV infection can occur without symptoms?	92 (23.8%)	294 (76.2%)	1.15 [0.700–1.888]	2.168* [1.336–3.519]
14	HPV causes genital (external organs of reproduction *e.g*., testis) warts?	102 (26.4%)	284 (73.6%)	0.836 [0.521–1.339]	2.298* [1.438–3.673]
15	HPV may cause other genital cancers (penis, anus)?	101 (26.2%)	285 (73.8%)	0.815 [0.505–1.315]	3.017* [1.860–4.896]

**Notes:**

Binary Logistic Regression was used, Ref: Gender = Female, Field = non-Health Sciences.

* *p* < 0.05 statistically significant.

**Table 3:** Respondents’ knowledge regarding human papillomavirus transmission (N = 386).

Q. No	Statement	Frequency	%
16	Health problems associated with Human papillomavirus		
	Cervical Cancer	148	38.8
	Penile Cancer	103	26.7
	Genital Warts	116	30.1
	HIV	57	14.8
	Don’t know	206	53.4
17	Prevention of Human papillomavirus		
	Practicing abstinence (avoiding sex)	121	31.3
	Vaccination	191	49.5
	Using Condoms	107	27.7
	Antibiotics	80	20.7
	Don’t know	261	67.6
18	Spread/transmission of Human papillomavirus		
	Cough or sneezing	63	16.3
	Genital skin-to-skin contact	177	45.9
	Contact with bodily fluids (blood)	121	31.3
	Don’t Know	217	56.2

**Notes:**

Multiple responses were selected by the respondents; therefore the sum of response may not be always 100%.

**Table 4:** Respondents’ knowledge and understanding about human papilloma virus vaccines.

Q.	Statement	Yes	No	Gender	Field
19	There is a vaccine that protects against HPV?	119 (30.8%)	267 (69.2%)	0.667 [0.429–1.038]	1.201 [0.778–1.854]
20	The HPV vaccine prevents the chances of cervical cancers?	147 (38.1%)	239 (61.9%)	0.506* [0.331–0.774]	0.863 [0.569–1.309]
21	Once vaccinated, women no longer have to be screened for cervical cancer?	69 (17.9%)	317 (82.1%)	1.700 [0.956–3.022]	1.404 [0.829–2.377]
22	The HPV vaccine is only for people who are sexually active?	55 (14.2%)	331 (85.8%)	1.266 [0.686–2.336]	2.578* [1.398–4.753]
23	The HPV vaccine should be given before the first sexual intercourse?	93 (24.1%)	293 (75.9%)	0.677 [0.419–1.092]	1.584* [1.150–2.990]

**Notes:**

Binary Logistic Regression was used, Ref: Gender = Female, Field = non-Health Sciences.

* *p* < 0.05 statistically significant.

**Table 5:** Recommendations for use of human papilloma virus vaccines (N = 386).

Q. No	Statement	Mean	Std. D	SA	A	N	D	SD	RI	Rank
24	If my friends knew about the HPV vaccine, they would approve/disapprove of me getting vaccinated against HPV.	2.626	2.367	75 (19.4%)	96 (24.9%)	148 (38.3%)	32 (8.3%)	35 (9.1%)	0.675	2
25	If my parents knew about the HPV vaccine, they would approve/disapprove of me getting vaccinated against HPV.	2.658	2.427	87 (22.5%)	77 (19.9%)	140 (36.3%)	45 (11.7%)	37 (9.6%)	0.668	3
26	If my doctor knew about the HPV vaccine, he/she would approve/disapprove of me getting vaccinated against HPV.	2.274	2.062	130 (33.7%)	89 (23.1%)	122 (31.6%)	21 (5.4%)	24 (6.2%)	0.745	1

**Notes:**

SA, Strongly Approve; A, Approve; N, Neutral; D, Disapprove; SD, Strongly Disapprove; RI, Relative Index.

### Ethical approval

The study was carried out according to the declaration of Helsinki. The ethical committee of the university approved the study protocol (Ethical Application Ref: H-2020-056 University of Hail, Saudi Arabia), where the Ethical Application Approval letter No: Nr.41/213/43602 is provided by the committee. The questionnaire had a pre-face that describes the nature and purpose of the study and a consent part that ensures anonymity and voluntary participation of participants.

## Data collection

Registered students from all colleges and departments at the University of Hail, Saudi Arabia, were approached regardless of their age, gender, marital status and educational status. The questionnaire was distributed to all the participants, and as an ethical prerequisite, verbal consent was obtained prior to participation in the study. The quality of the data was checked and revised before it was categorized into various variables. The missing data has been excluded while the included data is distributed as per the data type.

## Data analysis

The International Business Machines Corporation (IBM) Statistical Package for the Social Sciences (SPSS) version 20 was used to analyze data. Data were collected from the students through a completed survey questionnaire. Incomplete survey form were excluded from the analysis. Various variables was created in the SPSS to retrieve the data from the complete questionnaire forms to the SPSS software. The relationship between demographic and ordinal responses was assessed using binary and linear regression. Reference group were chosen among the variables which comes at the end and having a maximum number of participants among the dichotomous variables comes in the category of both demographics and ordinal responses chosen by the study participants (Ranganathan, Pramesh & Aggarwal, 2017). Dummy variables were created before the data analysis using binary logistic regression. Once the encoding of all the independent variables as dummy variables was done, the odd ratios and significance of the coefficients were calculated. The key factors that may affect the respondents’ opinions regarding HPV vaccination were identified using a relative importance index (RII) (Eq. (1)) (Elbarkouky, 2012). The item with the value closest to one RII value was rated as the most significant factor affecting the HPV reporting process (Gündüz, Nielsen & Özdemir, 2013).

(1) $$RII = \; \displaystyle{{\sum W} \over {A\; \times \; N}}\; ,$$

where *W* is the weight assigned to each element by the respondents, which varies from 1 to 5 (with 1 indicating ‘strongly disagree’ and 5 indicating ‘strongly agree’); *A* is the maximum weight (*i.e*., 5 in this case) and *N* is the total number of respondents. Regression analysis was then used to classify the variables influencing the respondents’ awareness of HPV vaccination, with gender (ref. male) and field of study (ref. non-health science) being covariates. When assessing the respondents’ answers, a statistically significant value of <0.05 was allocated.

## Results

A total of 477 students were approached. Of these, 386 students responded to the survey, with the response rate being 80%. The majority of the respondents (63%) were male, and 332 (86%) respondents were single among the study population. Most respondents were aged 21–25 years (75.6%), followed by 26–30 years (12.7%). Furthermore, most respondents (90%) were undergraduates. A total of 102 (26.6%) respondents were enrolled in pharmacy, 68 (17.6%) in education and 73 (18.9%) in other fields (various fields other than those listed), with lower participation (1.8%) being observed from the fields of physiotherapy and biological sciences (Table 1). Moreover, Bartlett’s test of sphericity used for assessing the content adequacy revealed that the contents were satisfactory and met the study requirements (Khan et al., 2016).

### Respondents’ knowledge regarding HPV

When asked about their general knowledge regarding HPV, 130 (33.7%) respondents reported that they had heard about HPV before. Approximately 174 (45.1%) respondents reported that HPV infections are rare in Saudi Arabia, 96 (24.9%) reported that HPV causes cervical cancer and 153 (39.6%) reported that HPV infects both genders. Furthermore, 102 (26.4%) respondents mentioned that HPV causes genital warts, almost the same number of respondents mentioned that HPV may cause other genital cancers (penile and anal) and approximately 29.5% of the respondents mentioned that HPV infection is a sexually transmitted disease. Most respondents (79%) were not convinced that the incidence of HPV is the highest among women in their 20s and 30s in Saudi Arabia. Moreover, nearly 76.2% of the respondents did not believe that HPV infection can occur without symptoms. Approximately 285 (73.8%) respondents thought that HPV does not cause genital warts, and almost the same number of respondents thought that HPV may cause anal and penile cancers. Regression analysis revealed that the gender of the respondents was not significantly associated with most questions in the section of knowledge regarding HPV; however, it was significantly associated with Q7 (odds ratio (OR) 0.566, confidence interval (CI) [0.360–0.891]). Moreover, the field of study was found to be significantly associated with most questions, except for Q9 (OR 0.825, CI [0.552–1.234]), Q11 (OR 1.472, CI [0.977–2.218]) and Q12 (OR 1.525, CI [0.929–2.505]) (Table 2).

### Respondents’ knowledge regarding HPV transmission

When asked about the health problems associated with HPV infection, 53.4% of the respondents stated that they did not know, 148 (38.8%) mentioned cervical cancer as a health problem associated with HPV infection, 116 (30.1%) mentioned genital warts, 103 (26.7%) mentioned penile cancer and 14.8% mentioned human immunodeficiency virus (HIV) infection. Regarding the mode of transmission of HPV, 217 (56.2%) respondents replied that they did not know, 177 (45.9%) stated that HPV spreads through genital skin-to-skin contact, 121 (31.3%) stated that it spreads through the exchange of bodily fluids and only 63 (16.3%) stated that it spreads through coughing or sneezing. Moreover, when asked about the preventive measures against HPV infection, while 261 (67.6%) respondents mentioned that they did not know, approximately 191 (49.5%) respondents mentioned vaccination as the preventive measure, 121 (31.3%) mentioned sexual abstinence, 27.7% mentioned the use of condoms and 20.7% mentioned the use of antibiotics (Table 3).

### Respondents’ knowledge regarding HPV vaccines

When asked about their understanding of the HPV vaccine, nearly 267 (62.2%) respondents mentioned that there is no vaccine for HPV and 239 (61.9%) mentioned that the HPV vaccine does not minimise the risk of cervical cancer. Approximately 82.1% of the respondents said that women do not need to be tested for cervical cancer after being vaccinated against HPV. Moreover, approximately 75.9% of the respondents believed that the HPV vaccine should be given before the first sexual encounter, whereas 331 (85.8%) stated that the HPV vaccine should only be given to sexually active adults. Regression analysis revealed that the gender of the respondents was not significantly associated with all the questions in the section of vaccine-related knowledge, except for Q20 (OR 0.506, CI [0.331–0.774]); however, the field of study was significantly associated with Q22 (OR 2.578, CI [1.398–4.753]) and Q23 (OR 1.584, CI [1.150–2.990]) (Table 4). Furthermore, the respondents reported that they would be far more likely to get HPV vaccines if recommended by their doctors (RII = 0.745), followed by their friends (RII = 0.675) (Table 5).

## Discussion

The present study assessed how multidisciplinary students at the University of Hail, Saudi Arabia, felt about HPV and what they knew about it. To our knowledge, this is one of the first such studies to be conducted in Hail. Except for a few questions, most respondents had borderline or poor knowledge regarding HPV. Despite being multidisciplinary students, roughly 66.3% of the respondents had never learned of HPV and only around 39.6% knew that HPV infects all genders. Most respondents were unaware that HPV causes cervical cancer, and nearly 45.1% were unaware that HPV infection is uncommon in Saudi Arabia. In a related study conducted in Pakistan (Khan et al., 2016), only 57% of students were aware of HPV, indicating that Saudi students are less knowledgeable about HPV. This lower level of knowledge may be attributed to the fact that non-health science students were more likely to participate in the present study. Similarly, almost 17.7% of participants in a study conducted at the University of Lagos, Nigeria, had learned of HPV and only 19.6% said that HPV causes cervical cancer (Nkwonta, 2018). Furthermore, the awareness of genital warts was lower in the present study than in a study conducted in Lahore, Pakistan; in that study, 52% of university students knew about genital warts (Khan et al., 2016). Inadequate information on HPV in various instructional materials used in cervical cancer awareness programmes in Saudi Arabia may be responsible for the lack of knowledge regarding genital HPV infection and genital warts. In the present study, more than 40% of the respondents knew that the HPV vaccine is not strictly meant for sexually active individuals. Furthermore, 38% of the respondents reported that HPV causes cervical cancer, 26.7% reported that it causes penile cancer, 30.1% reported that it causes genital warts and 14.8% reported that HIV infection is a health problem associated with HPV infection. These results were contradictory to those of a previous study on Columbian students conducted by Piñeros et al. (2013). In Saudi Arabia, there is a lack of information about HPV; therefore, some respondents in the present study thought that it can be avoided by using antibiotics. The present findings highlight the need for a well-designed educational programme on HPV infection in Hail, Saudi Arabia, where there is inadequate awareness of HPV infection and low self-perceived resistance to HPV infection and HPV-related diseases. Another significant finding of the present study is the respondents’ experience with HPV vaccination. The implementation of HPV vaccination is a big move forward, with approximately 49.5% of the respondents in the present study claiming that the vaccine would shield them from HPV. Surprisingly, a handful of respondents who were aware of the HPV vaccine mentioned that they would ask for their healthcare providers’ consent before getting vaccinated against HPV. In a related survey conducted in Italy, 81.7% of respondents mentioned that they would be able to get the HPV vaccine (Di Giuseppe et al., 2008); though a higher percentage of results was reported when compared with the current study results. The effect of HPV educational programmes and the national HPV immunisation programme, which provides free vaccines for girls aged 12 years, may explain the comparatively higher level of vaccine acceptance in Italy.

## Conclusion

This study may have policy implications and can serve decision-makers in that they should be aware of the overall level of knowledge among health-care workers and implement educational interventions to address information gap and assist health practitioners in consistently recommending the anti-HPV vaccine. The present findings revealed that respondents at the University of Hail, Saudi Arabia, had poor knowledge regarding the health issues associated with HPV, its preventive measures and its transmission. Nearly a quarter of the respondents were unaware of the availability of the HPV vaccine, while the rest had poor knowledge regarding the risks of recurrence following vaccination. Furthermore, more than 40% of the respondents were ignorant about the prevention of HPV, transmission and problems associated with HPV infection. However, 40% of the respondents said that they would get vaccinated against HPV if their parents approved it, while 56% said that they would get vaccinated if their doctor recommended it. Because both genders are the target of the HPV vaccine, training on HPV infection, related diseases, and prevention methods is required to support the critical role in the prevention of STIs, some of which are linked to the development of cancer. Universities provide a unique opportunity to communicate HPV knowledge to the target audience, and young adults see teachers and trusted clinicians as their preferred sources of information. Improving students’ understanding could have a positive impact on vaccine coverage rates.

## Strengths and Limitations

The present study is probably one of the initial studies to discuss HPV health literacy among Saudi students and to provide a clear understanding of students’ attitudes towards HPV. The findings can be used to increase HPV awareness by developing an effective educational strategy. However, the present findings cannot be applied to all students and young adults in Saudi Arabia. The data are more relevant to urban dwellers and trained adults. This study also might not be generalized on the overall population of the Hail region, as the sample population selected was the students of one of the main universities located in the region and it was chosen since it was the only public tertiary institution of the region. Furthermore, the present study did not assess the association between the respondents’ answers and their age and marital status. Further research with a larger sample size is recommended; such efforts would also aid in the development of educational services for various age ranges.

## Supplemental Information

10.7717/peerj.13140/supp-1 Supplemental Information 1 SPSS dataset.

10.7717/peerj.13140/supp-2 Supplemental Information 2 Research Questionnaire.

10.7717/peerj.13140/supp-3 Supplemental Information 3 Supplementary data for internal consistency of the questionnaire.

## Additional Information and Declarations

### Data Availability

The following information was supplied regarding data availability: The raw measurements are available in the Supplemental Files.
