# Supplementary material for: Knowledge, attitudes and perceptions regarding human papillomavirus among university students in Hail, Saudi Arabia
Source: PeerJ. 2022 Mar 23;10:e13140. doi: 10.7717/peerj.13140 (PMC8957278; doi:10.7717/peerj.13140)
Supplement: Supplemental Information 2 [file peerj-10-13140-s002.docx]

**Data collection tool Survey Questionnaire**

**Research Study Title: Knowledge, Attitudes, and Perception towards Human Papillomavirus among university students in Hail, Saudi Arabia**

**RESPONDENT DEMOGRAPHIC DATA**

| **Item no.** | **Demographics** | | | | |
| --- | --- | --- | --- | --- | --- |
| 1 | Gender | | | ○ Male | ○ Female |
| 2 | Age (Years) |  | Age group | ○ 18 – 30 ○ 31-40 ○ 41-50 ○ >50 | |
| 3 | Marital Status | ○ Single ○Married ○ Widow ○ Divorced | | | |
| 4 | Education | ○ Bachelors ○ Master ○ PhD ○ Medical Specialist | | | |
| 5 | Field of study | ○ Health Sciences ○ Non- Health Sciences | | | |
| 6 | Course Registered | ○ Pharmacy (Pharm-D) ○ MBBS ○ Biological Sciences  ○ Educational Studies ○ Business & Management Sciences  ○ Arts & Humanities ○ Social Sciences ○ Physical Sciences  ○ Bachelor of Dental Studies ○ Physiotherapy  ○ Others ________________________________ | | | |

**GENERAL KNOWLEDGE ABOUT HUMAN PAPILLOMAVIRUS**

| **Item no.** | **Statement** | **Response** | |
| --- | --- | --- | --- |
| 7 | Before taking this survey, had you ever heard of HPV (human papillomavirus)? | Yes | No |
| 8 | Is HPV sexually transmitted? | Yes | No |
| 9 | Are HPV infections rare in Saudi Arabia? | Yes | No |
| 10 | Does HPV cause cervical cancer? | Yes | No |
| 11 | Can HPV infect both men and women? | Yes | No |
| 12 | Is the incidence of HPV highest among women in their 20s and 30s? | Yes | No |
| 13 | Can a HPV infection occur without symptoms? | Yes | No |
| 14 | Does HPV cause genital (external organs of reproduction e.g. testes) warts? | Yes | No |
| 15 | Can HPV cause other genital cancers (penis, anus)? | Yes | No |

**KNOWLEDGE ABOUT HUMAN PAPILLOMAVIRUS**

| **Item no.** | **Statement** | **Response** |
| --- | --- | --- |
| 16 | Health problems associated with Human papillomavirus | ○ Cervical Cancer ○ Penile Cancer  ○ Genital Warts ○ HIV ○ Don’t Know |
| 17 | Prevention of Human papillomavirus | ○ Practicing abstinence (avoiding sex)  ○ Vaccination ○ By Using Condoms  ○ Antibiotics ○ Don’t Know |
| 18 | Spread/transmission of Human papillomavirus | ○ Cough or Sneezing  ○ Genital skin-to-skin contact  ○ Contact with body fluids (blood) ○ Don’t Know |

**RESPONDENTS KNOWLEDGE AND UNDERSTANDING ABOUT HPV VACCINES**

| **Item no.** | **Statement** | **Response** | |
| --- | --- | --- | --- |
| 19 | Is there is a vaccine that protects against HPV? | Yes | No |
| 20 | The HPV vaccine prevents the chances of cervical cancers | Yes | No |
| 21 | Once vaccinated, women no longer have to be screened for cervical cancer | Yes | No |
| 22 | The HPV vaccine is only for people who are sexually active | Yes | No |
| 23 | Should the HPV vaccine be given before commencing sexual intercourse? | Yes | No |

**RECOMMENDATION OF HUMAN PAPILLOMAVIRUS VACCINE**

| **Item no.** | **Statement** | **Response** |
| --- | --- | --- |
| 24 | If my friends knew about the HPV vaccine, they would approve of me getting vaccinated against HPV. | ○ Strongly Approve ○ Approve ○ Neutral  ○ Disapprove ○ Strongly Disapprove |
| 25 | If my parents knew about the HPV vaccine, they would approve of me getting vaccinated against HPV. | ○ Strongly Approve ○ Approve ○ Neutral  ○ Disapprove ○ Strongly Disapprove |
| 26 | If my doctor knew about the HPV vaccine, he/she would approve of me getting vaccinated against HPV | ○ Strongly Approve ○ Approve ○ Neutral  ○ Disapprove ○ Strongly Disapprove |
