# Supplementary material for: Knowledge, attitudes and perceptions regarding human papillomavirus among university students in Hail, Saudi Arabia
Source: PeerJ. 2022 Mar 23;10:e13140. doi: 10.7717/peerj.13140 (PMC8957278; doi:10.7717/peerj.13140)
Supplement: Supplemental Information 3 [file peerj-10-13140-s003.docx]

**Appendix 1.**

**Supplementary data for internal consistency of the questionnaire**

|  | **Scale mean**  **If item deleted** | **Scale variance if item deleted** | **Corrected item-total correlation** | **Cronbach's alpha if item deleted** |
| --- | --- | --- | --- | --- |
| 7. Before taking this survey, had you ever heard of HPV  (human papillomavirus)? | 19.01 | 53.765 | 0.645 | 0.870 |
| 8. Is HPV sexually transmitted? | 19.07 | 53.453 | 0.688 | 0.867 |
| 9. Are HPV infections rare in Pakistan? | 19.28 | 56.121 | 0.368 | 0.875 |
| 10. Does HPV cause cervical cancer? | 19.03 | 53.207 | 0.737 | 0.868 |
| 11. Can HPV infect both, men and women? | 19.02 | 53.831 | 0.648 | 0.867 |
| 12. Is the incidence of HPV is highest among women in their 20's and 30’s. | 19.26 | 54.899 | 0.533 | 0.873 |
| 13. Can a HPV occur without symptoms? | 19.26 | 54.798 | 0.549 | 0.868 |
| 14. Does HPV causes genital (external organs of reproduction e.g., testis) warts? | 19.06 | 53.019 | 0.758 | 0.864 |
| 15. Can HPV may cause other genital cancers (penis, anus)? | 19.09 | 53.704 | 0.663 | 0.866 |
| 16. Health problems associated with HPV. |  |  |  |  |
| a. Cervical cancer | 19.04 | 53.386 | 0.708 | 0.864 |
| b. Penile cancer | 19.31 | 55.608 | 0.461 | 0.872 |
| c. Genital warts | 19.17 | 54.121 | 0.621 | 0.871 |
| d. HIV | 19.46 | 57.857 | 0.189 | 0.875 |
| e. Don’t Know | 19.19 | 64.901 | -0.807 | 0.893 |
| 17. Prevention of Human papillomavirus |  |  |  |  |
| a. Practicing abstinence (avoiding sex) | 19.24 | 55.412 | 0.468 | 0.872 |
| b. Vaccination | 19.12 | 54.016 | 0.624 | 0.871 |
| c. By using Condoms | 19.33 | 55.624 | 0.494 | 0.872 |
| d. Antibiotics | 19.49 | 58.326 | 0.132 | 0.873 |
| e. Don’t Know | 19.14 | 64.901 | -0.796 | 0.896 |
| 18. Spread/transmission of Human papillomavirus |  |  |  |  |
| a. Cough or sneezing | 19.18 | 54.129 | 0.613 | 0.871 |
| b. Genital skin-to-skin contact | 19.47 | 57.884 | 0.187 | 0.877 |
| c. Contact with bodily fluids (blood) | 19.35 | 55.625 | 0.489 | 0.874 |
| d. Don’t Know | 19.55 | 58.319 | 0.129 | 0.877 |
| 19. Is there is a vaccine that protects against HPV? | 19.24 | 54.559 | 0.558 | 0.872 |
| 20. The HPV vaccine prevents the chances of cervical cancers. | 19.26 | 54.791 | 0.536 | 0.871 |
| 21. The HPV vaccine is only for people who are sexually active. | 19.43 | 56.938 | 0.302 | 0.876 |
| 22. Should the HPV vaccine be given before commencing sexual intercourse? | 19.14 | 53.704 | 0.667 | 0.868 |
| 23. Once vaccinated, women no longer have to be screened for cervical cancer. | 19.43 | 57.216 | 0.281 | 0.876 |
| 24. Friends knew about the HPV vaccine, they would approve of me getting vaccinated against HPV. | 16.21 | 49.404 | 0.541 | 0.875 |
| 25. If my parents knew about the HPV vaccine, they would approve of me getting vaccinated against HPV. | 16.12 | 48.956 | 0.535 | 0.876 |
| 26. If my doctor knew about the HPV vaccine, he/she would approve of me getting vaccinated against HPV. | 15.86 | 47.633 | 0.636 | 0.871 |
